# Epidemiological, clinical and biological features of malaria among children in Niamey, Niger

**DOI:** 10.1186/1475-2875-4-10

**Published:** 2005-02-09

**Authors:** Françoise Gay-Andrieu, Eric Adehossi, Véronique Lacroix, Moussa Gagara, Maman Laminou Ibrahim, Hama Kourna, Hamadou Boureima

**Affiliations:** 1 Laboratory of Medical Biology, National Hospital of Niamey, Niamey, Niger; 2 Department of Internal Medicine, B3, National Hospital of Niamey, Niamey, Niger; 3 Clinique Gamkalley, Niamey, Niger; 4 Université Victor Segalen, Bordeaux 2, Bordeaux, France; 5 CERMES (Centre de Recherches Médicales et Sanitaires), Niamey, Niger; 6 Department of Paediatrics B, National Hospital of Niamey, Niamey, Niger; 7 Department of Paediatrics A, National Hospital of Niamey, Niamey, Niger

## Abstract

**Background:**

Malaria takes a heavy toll in Niger, one of the world's poorest countries. Previous evaluations conducted in the context of the strategy for the Integrated Management of Childhood Illness, showed that 84% of severe malaria cases and 64 % of ordinary cases are not correctly managed. The aim of this survey was to describe epidemiological, clinical and biological features of malaria among <5 year-old children in the paediatric department of the National Hospital of Niamey, Niger's main referral hospital.

**Methods:**

The study was performed in 2003 during the rainy season from July 25^th ^to October 25^th^. Microscopic diagnosis of malaria, complete blood cell counts and measurement of glycaemia were performed in compliance with the routine procedure of the laboratory. Epidemiological data was collected through interviews with mothers.

**Results:**

256 children aged 3–60 months were included in the study. Anthropometrics and epidemiological data were typical of a very underprivileged population: 58% of the children were suffering from malnutrition and all were from poor families. Diagnosis of malaria was confirmed by microscopy in 52% of the cases. Clinical symptoms upon admission were non-specific, but there was a significant combination between a positive thick blood smear and neurological symptoms, and between a positive thick blood smear and splenomegaly. Thrombopaenia was also statistically more frequent among confirmed cases of malaria. The prevalence of severe malaria was 86%, including cases of severe anaemia among < 2 year-old children and neurological forms after 2 years of age. Overall mortality was 20% among confirmed cases and 21% among severe cases.

**Conclusions:**

The study confirmed that malaria was a major burden for the National Hospital of Niamey. Children hospitalized for malaria had an underprivileged background. Two distinctive features were the prevalence of severe malaria and a high mortality rate. Medical and non-medical underlying factors which may explain such a situation are discussed.

## Background

Malaria accounts for 1 in 5 of all children deaths in Africa. Late presentation, misdiagnosis, inadequate management, unavailability or stock-outs of effective drugs are factors that influence high case-fatality rates even among hospital in-patients [1]. One of the targets of the Roll Back Malaria (RBM) initiative [2] is to establish systems to guarantee that adequate stocks of drugs and clinical consumables are available to health facilities, and that health facility staff are trained and supervised for the rapid identification, resuscitation and subsequent clinical care of children with severe malaria. But many African countries are still a long way from meeting this objective, and it is admitted now that malaria disproportionally affects the poorest populations [3].

### Geographical and socio-economic situation

Niger is situated in the eastern part of West Africa in the Sahelo-Saharan zone. It is a landlocked country surrounded by Algeria, Libya, Chad, Nigeria, Benin, Burkina-Faso and Mali. The country's climate is a rainy season from June to October and a dry season during the rest of the year. The hottest months are April and May.

According to the Human Development Index (UNDP 2003), Niger ranks 174^th ^of 175 countries with an annual GDP of about $160 per capita (2001). The demographic situation is characterized by a population of 11,544,000 inhabitants (2002) which is expected to double by 2025 with an annual growth rate of 3.5% (1999–2002). The infant and child mortality rate is 255‰ and 63% of the population live below the poverty line ($1 per day per person) [4-6].

The Bamako Initiative of 1987 was adopted by Niger but in a context of extreme poverty, the generalization of the cost recovery may have an impact on the access to health facilities, which should be assessed.

### Malaria in Niger

Malaria is endemic in Niger with 97% of the population exposed to the risk of malaria [1], but some areas of the country are much more affected with the disease than others. Varying environmental conditions mean that the level of transmission will vary from one area to another: the northern part has an unstable situation while the South of the country (including Niamey, the capital), which is exposed to the annual monsoon rainfall following a long dry season [7,8] has an intermediate, stable situation. In the Sudan-Sahelian savanna, close to the River Niger, immunity and partial protection are acquired in children over 5 years of age [9]. *Plasmodium falciparum *is the predominant species partially associated with *Plasmodium malariae *and less frequently with *Plasmodium ovale*, according to geographical variations [7,8].

Among the anopheles of epidemiological importance, only *Anopheles gambiae *and *Anopheles arabiensis *may play a substantial role in malaria transmission but *Anopheles funestus *which was no longer found after 1970 seems to have reappeared in several sudano-sahelian zones of Niger [10,11].

Resistance to chloroquine was first described in 1991 [12] but few surveys have been conducted in the country so far [13,14]. Chloroquine remains as the first line treatment but such antimalarial treatment policy is now being challenged (2004).

Malaria remains a main cause of morbidity in Niger with an average of 850,000 cases per annum, corresponding to an annual incidence of 80‰ inhabitants, and 33.97% of reported deaths are due to malaria [4]. These estimates, however, lack accuracy as most of the reported cases are only "presumed cases" and many deaths do not occur at the hospital. It has also been estimated that only 50% of children under 5 with reported fever in the two previous weeks received chloroquine or any anti-malarial treatments, and less than 5% of households have insecticide-treated nets [1].

### Rationale

In Niger, as in many sub-Saharan countries, malaria takes a heavy toll, especially among young children in poor households. An evaluation – conducted in 2003 by the University Research Corporation [15] in the context of the strategy for the Integrated Management of Childhood Illness (IMCI) adopted by Niger in 1996 – showed that 84% of severe cases and 64% of ordinary cases are not correctly managed in national, regional and district hospitals.

A high proportion of deaths in the National Hospital of Niamey (NHN) is related to malaria. The poor quality of medical care makes it difficult to assess the real burden of malaria in terms of mortality and morbidity. Misdiagnosis is likely to be quite common, either in excess or default. The objective of the present prospective study was to describe the epidemiological, biological and clinical features of cases of malaria in children in the paediatric department of the NHN in order to recommend more relevant decision-making and interventions.

## Methods

### Study area

The study was carried out in the NHN during the rainy season of 2003 from July 25^th ^to October 25^th^, which is the peak transmission period. The NHN is the country's main referral hospital and has 852 beds. The paediatric department has two sections: section A (60 beds) for children from 0 to 24 months, and section B (60 beds) for children from 25 months to 15 years of age. A majority of patients come from the urban community of Niamey but some may be referred from all parts of the country.

### Patients and methods

Children from 3 months to 5 years of age (60 months) admitted to Paediatric department A or B for presumed malaria were included in the study and treated following procedures normally used in the department: children went through the emergency department where a clinical examination was performed by doctors or by medical students in their final year, venous blood was drawn for biological tests (for the study, laboratory tests were free of charge), confirmed cases of malaria were treated intravenously with quinine twice a day for 2–3 days, followed by oral treatment (WHO protocol adapted for Niger by the National Malaria Control Program).

Upon admission, or within 24 hours, a physician conducted interviews with mothers in order to collect epidemiological data. Information such as age of the parents, number of siblings, the child's rank in relation to siblings, care-seeking previously to hospitalization, delay between the onset of disease and admission to the NHN, and treatment before hospitalization, was collected. The socio-economic background of households was evaluated through the Niamey neighbourhood where children originated from, the educational level of the mother (whether illiterate or not), the occupation of the mother and father, and the existence or non existence of a regular salary.

Biological tests were performed along the routine procedures of the NHN's laboratory. Thick and thin blood smears were prepared and stained following standard procedures: Giemsa 10% for 20 minutes (Giemsa R, RAL, CML, Nemours, France) for thick smear, and rapid staining (RAL 555, CML) for thin smear. A thick smear was declared negative only after examination of 200 fields (obj × 100). All smears were checked the following day by a different technician. Parasitaemia was measured on thin smear and expressed as a percentage of parasitised red blood cells. Haematological tests – haemoglobin concentration, hematocrit and complete blood cell count – were performed automatically using a KX21 (Sysmex). The blood glucose rate was performed with a COBAS Mira (Roche) using the glucose oxidase technique (Biomérieux, Marcy l'Etoile, France).

According to the 2000 WHO criteria [16], severe malaria is defined as the presence of *P. falciparum *on thick smear and at least one of the following clinical or biological criteria : coma (Blantyre coma scale ≤ 2), impaired consciousness (Blantyre >2 and < 5), repeated convulsions (≥ 2/24 hours), prostration, respiratory distress, jaundice, metabolic acidosis (bicarbonates < 15 mmol/L), severe anaemia (Hb < 5 g/dL or Ht < 15%), hyperparasitaemia (parasitaemia > 4% in non-immune patients), macroscopic haemoglobinuria, renal failure, collapse (TAS < 60 mmHg before 5 years of age), abnormal bleeding or pulmonary oedema (X-ray criterion). The conditions in the NHN in 2003 made it impossible to asses acidosis, renal failure (based on creatinine) and pulmonary oedema. Only the following criteria could be considered: coma, impaired consciousness, convulsions, respiratory distress, jaundice, abnormal bleeding, severe anaemia, hyperparasitaemia, hypoglycaemia.

Children were weighed. The index weight-for-age (weight/age ratio) was calculated and analysed with the Epi-Info software, based on the reference population defined by the US National Center for Health Statistics.(NCHS). Malnutrition was defined as a weight/age ratio more than 2 SD below the NCHS's reference population [17]. Malnutrition was considered moderate between -2 and -3 SD and severe below 3 SD.

### Statistical methods

Version 6 of the program Epi-Info was used for statistical analysis. ANOVA and Kruskal Wallis tests were used for mean comparisons whenever appropriate. Proportions were compared using chi-square tests. Bivariate odds ratio and their 95% confidence intervals were calculated to measure the combination between fatal outcome and different variables. For all tests, a p-value below 0.05 was considered significant.

### Ethical issues

The routine management of the children was not changed. Verbal consent was obtained from the parents after informing them in their native language. The parents were allowed to remove their child from the study at any time during follow-up. All data was entered anonymously into a database and identification numbers were coded. No ethnic data was registered. The protocol was submitted to and approved by the Ministry of Public Health and Endemic Diseases Control's National Malaria Control Program.

## Results

### Population

256 children were recorded over the 3 month survey: 138 males, 118 females (sex ratio = 1.17). The average age was 20.2 months: 3–12 months, n = 103 (40%), 13–24 months, n = 103 (40%) and 25–60 months, n = 50 (20%). The average age of parents was 27 years for the mothers, 39 years for the fathers. The number of living siblings averaged 2.8, and 35% of the children were first born. All children came from the urban community of Niamey, and 241/256 (94 %) from poor neighbourhoods: unsanitary neighbourhoods, near the river or near permanent ponds, some of them without electricity or tap water. The educational level of the mothers was low: 246/256 (96%) were illiterate, and 248/256 (97%) were home workers. Only 58/256 fathers (23%) had a monthly fixed income (24 civil servants and 34 salaried workers). The others had unsteady jobs with no stable income.

77 children out of 217 documented files for this item (35%) were considered malnourished and 42% dehydrated as a result of clinical examination. The percentage of malnutrition, however, was 58% based on the weight/age ratio, of which 30% were moderate and 28% severe cases of malnutrition. The youngest children (under 2 years old), particularly the 13–24 months age group, which corresponds to the weaning period, were most affected (Table 1).

**Table 1:** Nutritional condition of children upon admission.

		3–12		13–24		24–60	
		
		number n = 103	%	number n = 103	%	number n = 50	%
Dehydration	severe	8	(7.8)	3	(2.9)	0	(0.0)
	mild	42	(40.8)	32	(31.1)	16	(32.0)
	absence	46	(44.7)	60	(58.3)	32	(64.0)
	ND	7	(6.8)	8	(7.8)	2	(4.0)
							
Clinical malnutrition^a^	severe	4	(3.9)	8	(7.8)	0	(0.0)
	mild	29	(28.1)	27	(26.2)	9	(18.0)
	absence	53	(51.5)	50	(48.5)	37	(74.0)
	ND	17	(16.5)	18	(17.5)	4	(8.0)
							
Weight / age ratio^b^	severe	26	(25.2)	33	(32.0)	4	(8.0)
	mild	30	(29.1)	32	(31.1)	6	(12.0)
	absence	39	(37.9)	30	(29.1)	16	(32.0)
	ND	8	(7.8)	8	(7.8)	24	(48.0)

^a^: clinically assessed malnutrition

^b^: malnutrition assessed through weight/age ratio

ND: not done

### Clinical and biological diagnosis of malaria

**Figure 1:**
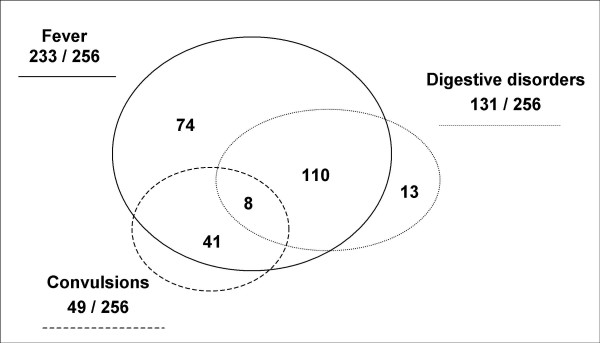
**Schematic representation of reasons for admission. **Number of cases showing each symptom against the total number of children admitted.

Upon admission, mothers reported fever (91% of cases), digestive disorder (51%) and convulsions (19%). These symptoms were often combined (Figure 1). The most common combination was fever + digestive disorders (43% of the admissions). Convulsions are always reported in a context of fever. The average time (as reported by parents) between the onset of disease and admission at the NHN was 5.6 days (in the 176 documented files) ranging from 1 (arrival at the hospital the same day) to 35 days. 54% of the children were brought to hospital within 3 days after the early symptoms, and 16% after 7 days.175 children (86%) of the 203 documented cases had already received a treatment before they were admitted to the NHN (home treatment or previous care in another health structure). We could not get accurate information on the kind of treatment they received, whether antimalaria, antibiotic, antipyretic or any other treatment. Parents did not know and most of the time had no written prescription, although 53% had a health book. Only the route of administration was precisely established: oral (49%), intramuscular (41%), intravenous (7%) or intrarectal (3%).

132/256 children admitted for presumed malaria (52%) had a positive thick blood smear. The percentage of treated children was the same among children with a negative thick blood smear as among those with a positive one (87% versus 86%). Both groups had quite a similar corrected axillary temperature (38.6° with positive thick smear versus 38.4°).

The clinical presentation of children admitted to hospital with a respective prevalence of the various observed symptoms is summarised in Table 2. Neurological symptoms – coma (Blantyre ≤ 2), impaired consciousness (Blantyre 3–4) or convulsions – were significantly more frequent when the thick smear was positive. Other unspecific symptoms (digestive disorders, dyspnea, hepatomegaly) were also more frequent with a positive blood smear although the difference was not significant. Only 3 cases of respiratory distress were observed, all three among children with a positive blood smear. The presence of splenomegaly was more frequent in children with a positive blood smear (26% versus 13%, p = 0.017).

**Table 2:** Prevalence of clinical symptoms and biological markers upon admission

	Positive blood smear		Negative blood smear		p value
	number/total	(%)	number/total	(%)	
**CLINICAL SYMPTOMS**					
coma^a^	22/77	(28,6)	7/64	(10,9)	p = 0,022
impaired consciousness^b^	15/77	(19,5)	11/64	(17,2)	
convulsions^c^	71/119	(59,7)	36/103	(35,0)	< 0,001
digestive disorders	58/93	(62,4)	63/104	(60,6)	NS
dyspnea	19/98	(19,4)	16/91	(17,6)	NS
respiratory distress	3/96	(3,1)	0/96	(0,0)	NS
hepatomegaly	36/107	(33,6)	28/95	(29,5)	NS
splenomegaly	28/106	(26,4)	12/93	(12,9)	0,017
					
**BIOLOGY**					
anaemia^d^	125/132	(94,7)	114/124	(91,9)	NS
severe anaemia^e^	55/132	(41,7)	39/124	(31,5)	NS
hypoglycaemia^f^	30/132	(22,7)	21/124	(16,9)	NS
thrombopaenia^g^	93/132	(70,5)	43/124	(34,7)	<0,001

^a^: Blantyre score ≤ 2

^b^: Blantyre score = 3–4

^c^: ≥ 2 / 24 hours

^d^: haemoglobin < 11 g/dL

^e^: haemoglobin < 5 g/dL

^f^: glyceamia < 2.2 mmol/L

^g^: platelets < 150 000 / mm3

The haemoglobin rate in the overall population was 6.5 g/dL (6.17 with positive blood smear, 6.94 with negative blood smear, p = 0.038). 94 % of children had anaemia (haemoglobin < 11 g/dL). This massive proportion of anaemia was observed in both groups: 95% versus 92% (NS) for "simple anaemia" (haemoglobin < 11 g/dL), and 42% versus 31% (NS) for severe anaemia (haemoglobin < 5 g/dL) (Table 2). Similarly, hypoglycaemia was more frequent with a positive blood smear but the difference was not significant (23% versus 17%, NS) (Table 2). The average of platelets was 141,000 in children with a positive blood smear, and 292,000 for those with a negative blood smear (p < 0.001). Based on a definition of thrombopaenia as a number of platelets below 150000/mm^3^, we found that thrombopaenia was statistically combined with a positive blood smear (70% versus 35%, p < 0.001) (Table 2). A similar result (p < 0.001) was obtained with the cut off = 100 000/mm^3^.

### Severe malaria attacks

**Figure 2:**
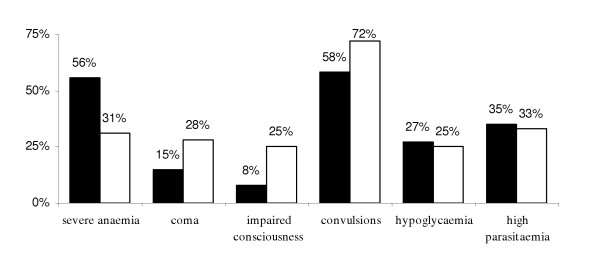
**Prevalence of severe malaria criteria by age group. **Prevalence (%) of different WHO criteria among severe cases of malaria in children by age: 3–24 months (black) and 25–60 months (white)

114/132 children with a positive blood smear (86%) met the criteria of severe malaria according to the clinical and biological criteria explored in this study. The most frequent criteria were convulsions (71/114 = 62%) and severe anaemia (55/114 = 48%) (Table 3). Some children were affected with combined criteria: 24/114 (21%) showed impaired consciousness and convulsions, 28/114 (25%) showed a neurological form (coma, and /or impaired consciousness and/or convulsions) and severe anaemia. No collapse, jaundice or spontaneous bleeding were recorded. Total mortality in the studied population was 17%, with a higher -although not significant (20% versus 13%) – rate among individuals with a positive blood smear. Mortality among children with severe malaria was 21%. Table 3 shows specific case fatality rate according to observed criteria of severe malaria. Only hypoglycaemia and coma were related to a higher mortality. Clinical presentation of severe malaria was analysed in terms of age. Figure 2 shows that severe anaemia is more frequent among children of less than 24 months (56% versus 31 %) and, conversely, the neurological forms are more frequent after 24 months of age. The prevalence of hypoglycaemia and high parasitaemia were similar in the two age groups.

**Table 3:** Prevalence of severity criteria among severe malaria cases with respective case fatality rate and relative risk of dying

	prevalence		case fatality rate		Odd Ratio	p value
			
	number n = 114	%	number/total	%	95 % IC	
coma^a^	22	(19.3)	12/21	(57.1)	9.33 (2.85 – 31.58)	< 0.001
impaired consciousness^b^	15	(13.2)	1/14	(7.1)	0.26 (0.01 – 2.07)	NS
convulsions^c^	71	(62.3)	18/71	(25.3)	4.42 (0.88 – 29.83)	0.04
respiratory distress	3	(2.6)	3/3			0.002
severe anaemia^d^	55	(48.2)	11/50	(22.0)	1.10 (0.40 – 3.05)	NS
hypoglycaemia^e^	30	(26.3)	11/28	(39.3)	3.72 (1.26 – 11.05)	0.006
high parasitaemia^f^	39	(34.2)	7/37	(18.9)	0.82 (0.27 – 2.42)	NS

^a^: Blantyre score ≤ 2

^b^: Blantyre score = 3–4

^c^: ≥ 2 / 24 hours

^d^: haemoglobin < 5 g/dL

^e^: glycaemia < 2.2 mmol/L

^f^: parasitaemia > 4%

## Discussion

### Description of the population

Hospitalized children in NHN were from underprivileged families: destitute neighbourhoods and disadvantaged socio-economic groups. This was confirmed by their poor nutritional conditions. 58% of the children had a weight/age ratio below 2 SD. This percentage is higher than national results previously published: the percentage of children with a weight/age ratio below 2 SD was 36% in 1992, and 50% in 1998 [18]. The delay before admission (5.6 days – ranging 1 to 35) is high but quite similar to other studies conducted in Africa: 6 days (ranging 1 – 30) in Dakar [19], 3.1 days (ranging 0–61) in Ouagadougou among urban patients [20]. One of the consequences of such a delay is that patients arrive at an advanced stage of disease. But in most cases, the NHN was not the first form of care sought. Interviews with the mothers highlighted the fact that traditional beliefs are still deeply rooted in families, and they interfere with the care-seeking pattern. For example malaria, "hemar ize" (= "the product of harvest season" in Zarma language) is still considered to be caused by "the smell of new plants during the rain season", despite the fact that there is some understanding of the part played by the mosquito as a result of health education. Convulsions are often linked to popular nosological entities, either "humburukumey" ("fear" in Zarma) whose origin is attributed to djins or witches, or "kyura" ("bird" in Zarma), i.e. "a bird having flown over the pregnant woman and dropped some graveyard soil onto her" (JP Olivier de Sardan, personal communication). This idea of a bird being responsible for children's convulsions is common to most of African societies and is beyond the medical context of malaria [21]. The occurrence of these convulsions, despite the parent's worry, does not necessarily encourage them to apply for a "modern" medical structure, but to refer more frequently to a "traditional" type of medicine or even to religious or superstitious practices. Thus, the neurological signs of malaria are identified and cured without mentioning malaria. The consequences can be serious, leading to delays in diagnosis and use of inefficient or even harmful traditional treatments. When finally reaching the hospital, because of the requirement for cost recovery, parents cannot afford all the medical examinations or treatments which should be undertaken.

### Malaria at NHN

Diagnosis was confirmed by thick blood film in 52% of cases which shows that clinical diagnosis is not accurate and needs to be confirmed by microscopic examination. This range of 50% of confirmed malaria attacks has already been observed 20 years ago in quite a similar health situation [22]. The clinical presentation was unspecific, but a significant combination of positive blood smear with neurological symptoms, and with splenomegaly, was observed. From a biological point of view, only thrombopaenia was significantly found to be combined with a positive blood smear. This importance of splenomegaly [23,24] and thrombopaenia [25,26] in the diagnosis of malaria has already been described and discussed in previous studies.

With 94% of anaemic children, the "anaemia" symptom is not typical of malaria in this population. Most children must have had one or several previous attacks of malaria during the transmission season even if their test is negative on arrival at the hospital. It would be interesting to assess the impact of treatments carried out before hospitalization (frequent, but accurate reporting is problematic as mentioned above) on the biological diagnosis. Non-specific clinical diagnosis followed by a presumptive treatment carried out without any biological confirmation, with an almost total absence of written follow-up in the management of children, are elements showing how difficult it is to diagnose malaria attacks in endemic areas. Both theoretical and practical parameters interfering in the diagnosis of malaria have been reviewed by Rogier [27].

### Severity of malaria at NHN

A particularly high percentage of severe malaria cases (86 %) was noted in the study population. Five children aged 6 months or under (one of them aged 3 months) were suffering from severe malaria, confirming the existence of severe malaria among very young children as already pointed out in other studies [28,29]. The new WHO criteria increase the percentage of severe cases [30]. This percentage may have been underestimated in this survey as some criteria could not be included for practical reasons. One of the problems was the difficulty of taking prostration into account: even if the WHO definition is clear (inability to sit upright for a child normally able to do so, or to drink in the case of children too young to sit) [16] this criterion could not be retained because of subjective interpretation. The absence of systematic chest X-ray and systematic creatinine measurements is unlikely to modify the results significantly as other surveys showed that pulmonary oedema and renal failure are infrequent criteria of severity among children [19,20]. The impossibility of performing blood gas analysis was more problematic since the importance of acidosis has been pointed out in several studies [16,31,32]. Consequently two major clinical presentations were observed: neurological form (coma, impaired consciousness or convulsions) and severe anaemia. Different breakdowns by age of these clinical presentations with a strong predominance of severe anaemia among 0–2 year-old children were reported. Several studies have already described links between clinical presentation, age of patients, and transmission level [19,20,33]. In Niamey, the severity of anaemia is probably enhanced by the very precarious nutritional condition of the infant population. Other severity criteria in the study population were hypoglycaemia and hyperparasitaemia. The small number of cases of respiratory distress (3/114 severe cases) is surprising compared to other studies [19,34], but the 3 children showing a respiratory distress in Niamey died. One may surmise that some health workers are not always sufficiently trained in assessing the severity of patients. The dramatic lack of therapeutic facilities in this hospital, and more specifically the insufficiencies of the intensive care service (5 intensive care beds for a 800-bed hospital, none of which is designated for paediatric resuscitation), does not encourage adequate concern in medical workers to make an accurate assessment based on severity criteria.

### Mortality

The overall case fatality rate of malaria was 20% among confirmed cases and 21% among severe cases. These values are higher than those observed in other African countries also based on 2000 WHO criteria: 11.9% in Ethiopia [35], 12% in Senegal [30], and 14.58% in Madagascar [28]. The first line treatment in Niger remains chloroquine but in the hospital, according to WHO recommendations, children were treated intravenously with quinine twice a day for 2–3 days, followed by oral treatment (WHO protocol adapted for Niger by the National Malaria Control Program).

However, such a level of hospital mortality among children who have already received treatment before admission could indicate an increase in the level of chloroquine resistance. Data on resistance is still insufficient in Niger but studies are presently being carried out. Detection of the *pfcrt *(T76) mutation which confers chloroquine resistance [36] was performed on 30 isolates among the 114 severe cases. The *pfcrt *mutation was present in 10/30 samples (ML Ibrahim, personal communication). These preliminary results need to be completed but such a level of genotypic drug resistance clearly seems insufficient to explain the high mortality rate observed at the NHN. Other implied factors have to be identified.

One of them is anaemia. As the prevalence of severe anaemia is very high, the capacity of the blood bank to provide blood in a short time is an important factor in reducing mortality. Transfusion facilities are woefully inadequate in the NHN.

## Conclusions

This survey conducted at the National Hospital of Niamey during the 2003 rainy season was an opportunity to examine the features of childhood malaria in a referral hospital of one of the world's lowest income countries. One of the dominant features in the NHN is that malaria affects an underprivileged population already affected by malnutrition. Only 52% of presumed cases are parasitologically confirmed. The rate of severe cases is high (86%) with two main clinical presentations: severe anaemia in less than 2-year old children and neurological form between 2 and 5-year old children. The mortality rate (21% among severe cases) is higher than established by most previously published data.

The study highlights several underlying factors that contribute to such an alarming situation:

(1) Patient factors: poor socio-economic background leading to multiple difficulties in getting access to healthcare (e.g. transport fees, hospital admission fees, biological tests, drug treatments...), and also the persistence of traditional beliefs interfering with care seeking behaviour.

(2) Provider factors: the recovery of medical care costs, although essential for the financial survival of the hospital, leads to multiple difficulties for the patient as seen above. Healthcare quality standards are also too low at all levels of the health organization, not just because of technical deficiencies (e.g. deficiency in intensive care units or in transfusion supplies) but also because of the inadequate training and attitudes of health-care workers.

All these factors need to be taken into account in order to find ways of improving the management of malaria in children. A recent awareness initiative is under way at the NHN, and several initiatives are being taken towards a gradual involvement of the hospital in a quality-oriented policy. But such local interventions will not reach their goal if extra efforts are not made at all levels to fight against disadvantages in the access to health facilities.

## Authors' contributions

FGA was an initiator of the study and took part in its development, supervised laboratory staff, took part in the analysis of the data, and wrote this article. EA was an initiator of the study, took part in its development, coordinated the clinical study, took part in the analysis of the data and in the writing of the paper. VL and MG took part in the development of the study, collected clinical and epidemiological data, and analysed the data. MLI conducted molecular tests in the Institut Pasteur de Madagascar. HK and HB supervised medical and nursing staff in the paediatric department. All authors have read and approved the final manuscript.
